# Transcriptional Regulation of Acyl-CoA:Glycerol-*sn*-3-Phosphate Acyltransferases

**DOI:** 10.3390/ijms20040964

**Published:** 2019-02-22

**Authors:** Ken Karasawa, Kazunari Tanigawa, Ayako Harada, Atsushi Yamashita

**Affiliations:** 1 Laboratory of Molecular Pharmaceutics, Faculty of Pharma-Sciences, Teikyo University, Tokyo 1738605, Japan; tanigawa@pharm.teikyo-u.ac.jp (K.T.); harada-a@pharm.teikyo-u.ac.jp (A.H.); 2 Laboratory of Biological Chemistry, Faculty of Pharma-Sciences, Teikyo University, Tokyo 1738605, Japan; ayamashi@pharm.teikyo-u.ac.jp

**Keywords:** GPAT, TAG, SREBP-1c, PPARγ, insulin resistance, obesity, tumorigenesis

## Abstract

Acyl-CoA:glycerol-*sn*-3-phosphate acyltransferase (GPAT) is an enzyme responsible for the rate-limiting step in the synthesis of glycerophospholipids and triacylglycerol (TAG). The enzymes of mammalian species are classified into four isoforms; GPAT1 and GPAT2 are localized in the mitochondrial outer membrane, whereas GPAT3 and GPAT4 are localized in the endoplasmic reticulum membrane. The activity of each enzyme expressed is associated with physiological and pathological functions. The transcriptional regulation is well known, particularly in GPAT1. GPAT1 mRNA expression is mainly regulated by the binding of the transcriptional factor SREBP-1c to the specific element (the sterol regulatory element) flanking the GPAT1 promoter. The TAG level is controlled by the insulin-induced transcriptional expression of GPAT1, which occupies most of the GPAT activity in the liver. The transcriptional regulation of the other three GPAT isoforms remains undetermined in detail. It is predicted that retinoic acid serves as a transcription factor in the GPAT2 promoter. PPARγ (peroxisome proliferator-activated receptor γ) increases the mRNA expression of GPAT3, which is associated with TAG synthesis in adipose tissues. Although GPAT has been considered to be a key enzyme in the production of TAG, unexpected functions have recently been reported, particularly in GPAT2. It is likely that GPAT2 is associated with tumorigenesis and normal spermatogenesis. In this review, the physiological and pathophysiological roles of the four GPAT isoforms are described, alongside the transcriptional regulation of these enzymes.

## 1. Introduction

**Figure 1:**
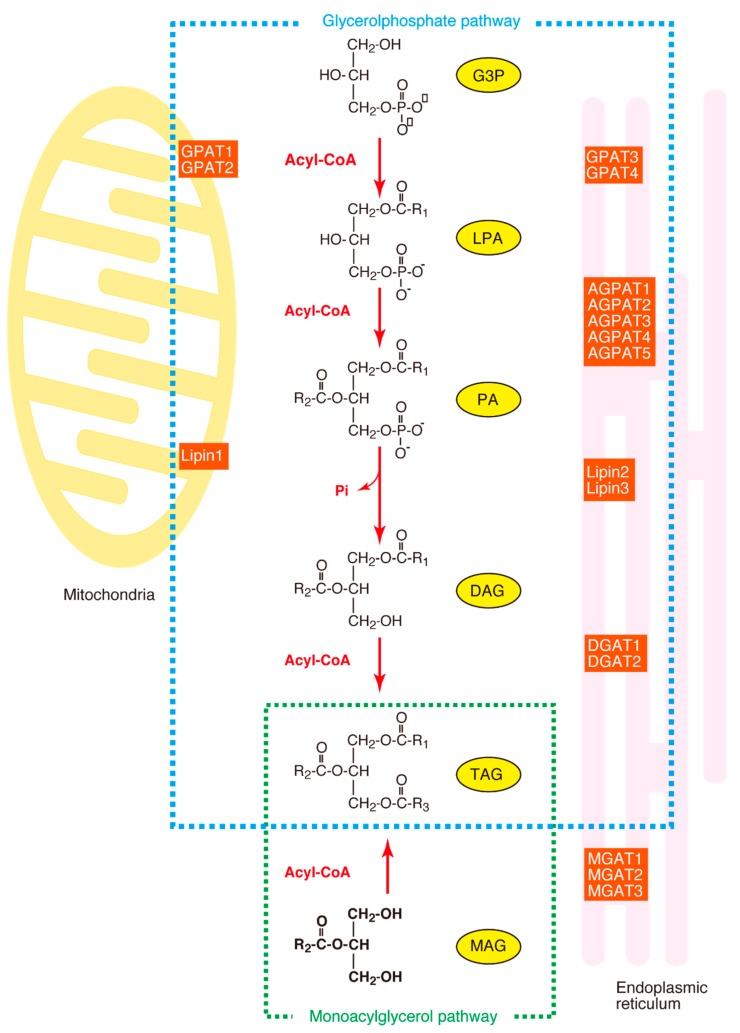
The synthesis of triacylglycerol (TAG). The glycerol phosphate pathway and the monoacylglycerol pathway are surrounded by dotted lines. MAG, monoacylglycerol; MGAT, monoacylglycerol acyltransferase; DHAP, dihydroxyacetone phosphate; DHAPT, dihydroxyacetone phosphate acyltransferase; DHAP-OR, dihydroxyacetone phosphate oxido-reductase; G3P, glycerol-3-phosphate; LPA, 1-acyl-G3P; PA, 1, 2-diacyl-G3P; DAG, diacylglycerol; GPAT, acyl-CoA:glycerol-*sn*-3-phosphate acyltransferase; DGAT, diacylglycerol acyltransferase; Lipin, phosphatidic acid phosphatase.

The acylation of glycerol-3-phosphate (G3P) is the first step, as well as the rate-limiting step, in the synthesis of glycerophospholipids and triacylglycerol (TAG). The enzyme which catalyzes this reaction is designated acyl-CoA:glycerol-*sn*-3-phosphate acyltransferase (GPAT) (EC 2.3.1.15). TAG is synthesized in several steps from the glycerol-3-phosphate (G3P) in most tissues (the glycerol phosphate pathway), as shown in Figure 1, although another synthetic pathway (the monoacylglycerol pathway) is dominant in the small intestine. In the glycerol phosphate pathway, G3P is acylated by GPAT to form LPA (1-acyl-G3P, abbreviated to AGP). LPA is esterified by acyl-CoA:AGP acyltransferase (AGPAT) to form PA (1, 2-diacyl-G3P), which is subsequently hydrolyzed by phosphatidic acid phosphatase (PAP, also known as Lipin) to form DAG (diacylglycerol). Finally, DAG is esterified by DAG acyltransferase (DGAT) to form TAG. In this glycerol phosphate pathway, the GPAT is the rate-limiting enzyme because it shows the lowest specific activity among the enzymes involved in TAG synthesis [1]. 

In mammals, four GPAT isoforms have been identified. Among these enzymes, GPAT1 and GPAT2 are localized in the mitochondrial outer membrane, whereas GPAT3 and GPAT4 are localized in the endoplasmic reticulum membrane [2]. Many studies focus on the regulation of the activities of these enzymes because the accumulation of TAG is closely related to the development of chronic metabolic diseases, including obesity, insulin resistance, and type 2 diabetes mellitus (T2DM) [3,4]. In particular, the mechanism of gene expression has been clarified in GPAT1 and the transcriptional factors involved in the activation of the promoter of the GPAT1 gene have been identified. In recent years, unexpected functions have been found in these enzymes, including their involvement in tumorigenesis and spermatogenesis [4]. In addition to the regulatory mechanism of these enzymes, their physiological and pathophysiological functions are described in this review. 

## 2. Characteristics of GPAT Isoforms

### 2.1. Classification of GPAT Isoforms

**Figure 2:**
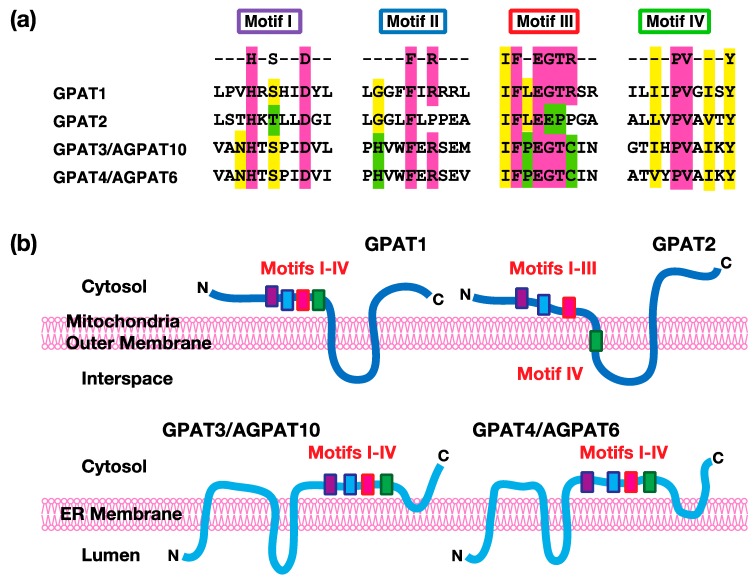
The acyltransferase motifs and predicted transmembrane topology in acyl-CoA:glycerol-*sn*-3-phosphate acyltransferases (GPATs). (**a**) The conserved amino acid residues of the acyltransferase motifs are shown in color. (**b**) The predicted transmembrane segments and their relationship to the acyltransferase motifs of GPATs. The transmembrane segments of each enzyme were predicted by several algorithms, including TMHMM and SOSUI. The locations of the acyltransferase motifs I–IV have also been depicted. The membrane is the mitochondrial outer membrane for GPAT1 and GPAT2 or the endoplasmic reticulum (ER) membrane for GPAT3/AGPAT10 (acyl-CoA:AGP acyltransferase10) and GPAT4/AGPAT6 (acyl-CoA:AGP acyltransferase6), respectively. The top or bottom sides of the membrane show the cytosol or interspace/lumen. In GPAT1, GPAT3, and GPAT4, all of the acyltransferase motifs (I, II, III, and IV) are predicted to be located in the cytosolic side of the membranes. In GPAT2, the motifs I, II, and III are also predicted to be located in the cytosolic side, while motif IV is in the first transmembrane segment. The membrane topology of GPAT1 and GPAT2 was also confirmed by the experiments [7,8].

At present, four kinds of GPAT isoforms have been identified. Among them, GPAT1 and GPAT2 are localized in mitochondrial outer membranes, whereas GPAT3 and GPAT4 are localized in endoplasmic reticulum membranes. Mitochondrial GPAT isoforms account for about 10% of the total GPAT activity in most tissues, except the liver, and microsomal GPAT isoforms account for the remaining total activity [1]. All four of these enzymes have structurally conserved motifs (I–IV) involved in catalytic reactions [5,6]. As shown in Figure 2, Motif I includes conserved His and Asp. These residues act as a base to remove a proton from the hydroxyl group at the *sn*-1 position of G3P to facilitate a nucleophilic attack on the acyl-CoA. Motif II includes conserved Phe and Arg. These residues are involved in the binding of the acyl acceptor G3P. Motif III includes conserved Phe, Glu, Gly, Thr, and Arg. These residues are important for the binding of the acyl acceptor G3P. Motif IV includes hydrophobic amino acids, such as Pro. This motif participates in the binding of the acyl donor acyl-CoA. These GPAT isoforms are distinguished by their sensitivity to sulfhydryl reagents, like N-ethylmaleimide (NEM), and their subcellular localizations (mitochondrial vs. microsomal), as described in the following sections. 

### 2.2. GPAT1

Mitochondrial GPAT1 was the first cloned enzyme in mammals [9]. Human GPAT1 contains 828 amino acids and its molecular weight is 94 kDa. The GPAT1 protein was purified from the Sf9 insect cells infected with the recombinant virus, containing the mouse GPAT1 gene [10] and rat liver mitochondria [11]. The addition of exogenous phospholipids is required to reactivate the enzyme activity of both preparations because enzyme activity is lost in the process of purification. The amino and carboxyl termini of GPAT1 face the cytosol. Conserved acyltransferase motifs (I–IV) are located in the portion close to the amino terminus and are followed by two transmembrane domains with a stem-loop which faces the transmembrane space [12]. Among the four GPAT isoforms, only GPAT1 is resistant to *N*-ethylmaleimide (NEM)-induced inhibition [1]. GPAT1 prefers saturated acyl-CoA, such as palmitoyl-CoA, and selectively transfers acyl-CoA to the *sn*-1 position of G3P [6]. This enzyme is highly expressed in lipogenic tissues, such as the liver and adipose tissues. Although the proportion of the GPAT1 activity in the total activity is substantially low, GPAT1 activity in the liver accounts for 30%–50% of the total GPAT activity. Therefore, GPAT1 contributes to the synthesis of TAG, particularly in the liver.

### 2.3. GPAT2

Another mitochondrial isoform, GPAT2, was first identified in GPAT1^−/−^ mice [13]. Human GPAT2 contains 801 amino acids and its molecular weight is 89 kDa. The amino acid similarity between human GPAT1 and GPAT2 is 32.3%. Similarly to GPAT1, GPAT2 has two transmembrane segments, with the amino and carboxyl termini facing the cytosol. Conserved acyltransferase motifs (I–III) are located in the portion close to the amino terminus, whereas motif IV is within the membrane [8]. The tissue expression and biochemical properties of GPAT2 are quite different from GPAT1. The GPAT2 mRNA expression is 50-fold higher in the testes than in other tissues and the GPAT2 mRNA is not altered by fasting or refeeding [14]. Given that the other GPAT isoforms, including GPAT1, GPAT3, and GPAT4, are highly expressed in lipogenic tissues, it is likely that GPAT2 plays a role in something other than the regulation of lipogenesis. In contrast to GPAT1, GPAT2 is sensitive to NEM and prefers arachidonoyl-CoA as the acyl donor [14,15].

### 2.4. GPAT3

The microsomal isoform GPAT3 is also designated as GPAT3/AGPAT10 because the cDNA of GPAT3 is identical to that cloned as AGPAT10 [16]. This enzyme shows both GPAT and AGPAT activities. Human GPAT3 contains 434 amino acids and its molecular weight is 49 kDa. This isoform possesses at least two transmembrane segments, with the amino and carboxyl termini facing the cytosol. Different from GPAT1 and GPAT2, conserved acyltransferase motifs (I–IV) reside in the carboxyl terminus side of the second transmembrane segment. This enzyme is sensitive to NEM. GPAT3 shows catalytic activity towards a broad range of saturated and unsaturated long-chain fatty acyl-CoA, such as palmitoyl-CoA, oleoyl-CoA, and linoleoyl-CoA [17]. The acylation of arachidonoyl-CoA to G3P is low. Sukumaran et al. have reported that the expression of GPAT3 mRNA is most abundant in adipose tissue, followed by the testes and kidney in humans [16]. Cao et al. have reported that the expression of GPAT3 mRNA is significantly lower in the adipose tissue, as compared to the kidney, heart, muscle, and testes [17]. However, given that the microsomal GPAT activity is dramatically increased 30- to 100-fold during adipocyte differentiation [18] and the total GPAT activity of white adipose tissue (WAT) is reduced by 80% in GPAT3^−/−^ mice, it is likely that GPAT3 plays an important role in TAG synthesis in WAT [19].

### 2.5. GPAT4

Another microsomal isoform, GPAT4, was originally designated as AGPAT6 because the cDNA was cloned based on the homology of its amino acid sequences of AGPAT1 and AGPAT2. However, this enzyme was renamed GPAT4 because it was demonstrated that this enzyme possessed GPAT activity but not AGPAT activity [13]. Human GPAT4 contains 456 amino acids and its molecular weight is 52 kDa. The amino acid similarity between human GPAT3 and GPAT4 is 68.9%. Both the amino and carboxyl terminal domains face the cytosol. It is predicted that a hairpin sequence formed by two hydrophobic helices is embedded within the endoplasmic reticulum (ER) membranes [20]. Similarly to GPAT3, conserved acyltransferase motifs (I–IV) reside in the carboxyl terminal side of the second transmembrane segment. GPAT4 is sensitive to NEM. GPAT4 shows catalytic activity towards both saturated and unsaturated long-chain acyl-CoA, such as palmitoyl-CoA, oleoyl-CoA, and linoleoyl-CoA, but the activity towards stearoyl-CoA is low [21]. GPAT4 is broadly expressed within various human tissues [21]. The NEM-sensitive GPAT activity is remarkably reduced in brown adipose tissue (BAT), but not in WAT in GPAT4 ^−/−^ mice, as compared with wild-type mice [22]. This observation suggests that GPAT4 plays an important role in TAG synthesis in BAT. On the other hand, GPAT4 is highly expressed in mammary gland epithelium which is involved in milk production. The amounts of DAG and TAG in the milk from GPAT4 ^−/−^ mice are reduced by 90%, as compared with wild-type mice. As a result, the offspring from GPAT4 ^−/−^ female mice die within 48 h, unless they are transferred to foster mothers [23]. Thus, GPAT4 is required for TAG production in mammary glands.

## 3. Regulation of GPAT Isoforms

### 3.1. Transcriptional Regulation

Among the four kinds of GPAT isoforms, GPAT1 expression is controlled by a transcription factor-designated sterol regulatory element-binding protein, (SREBP)-1c [1]. SREBP-1c is a member of the sterol regulatory element-binding proteins (SREBPs), which belong to the basic helix-loop-helix leucine zipper family. SREBPs are produced as membrane-bound precursors and proteolytic cleavage releases the mature amino-terminal domain into the nucleus [24]. This domain binds to a classical sterol regulatory element (SRE) sequence (ATCACCCCAC) or SRE-like sequences in the enhancer region of the target genes to activate transcription [25]. SREBP-1a and SREBP-1c are produced by a single gene located on human chromosome 17p11.2, whereas SREBP-2 is produced by a separate gene located on human chromosome 22q13. SREBP-1 is responsible for the nutritional induction of hepatic lipogenic enzymes, whereas SREBP-2 regulates cholesterol synthesis [26]. On the other hand, SREBP-1c is also transcriptionally activated by the binding of the heterodimer consisting of a liver X receptor (LXR) and retinoid X receptor (RXR) to LXR-responsive elements (LEREs) located in the upstream part of the SREBP-1c gene [27,28]. 

**Figure 3:**
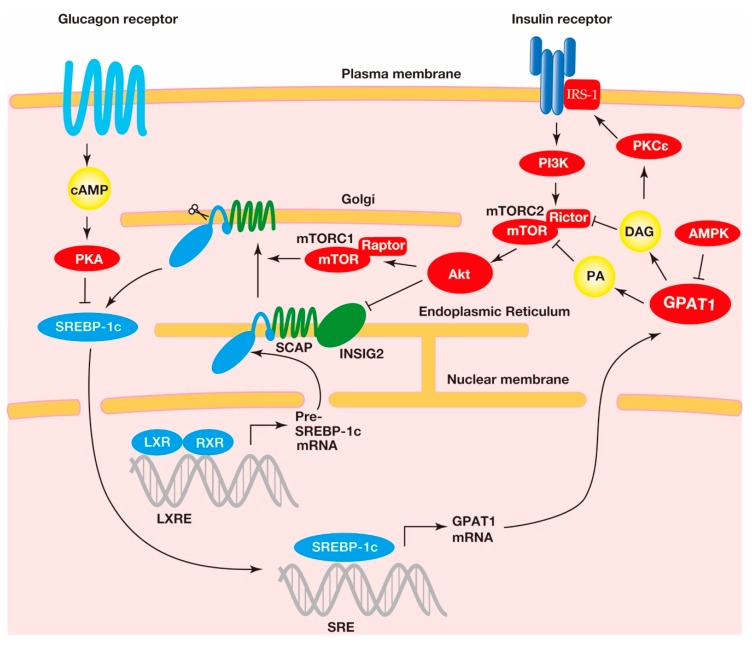
The regulation of the expression and cleavage of sterol regulatory element-binding protein-1c (SREBP-1c) and the transcriptional activation of the acyl-CoA:glycerol-*sn*-3-phosphate acyltransferase1 (GPAT1) gene. A heterodimer consisting of the liver X receptor (LXR) and retinoid X receptor (RXR) binds to the SREBP-1c precursor gene promotor region at the LXR response element for transcriptional activation. The SREBP-1c precursor resides at the endoplasmic reticulum membrane with the SREBP cleavage-activating protein (SCAP) and insulin-induced gene (INSIG) proteins. The complex, consisting of the SCAP and the SREBP-1c precursor, translocates to the Golgi, where the SREBP-1c precursor undergoes proteolytic cleavage. This translocation is involved in the mechanistic target of rapamycin complex 1 (mTORC1)-mediated indirect activation of protein kinase B (Akt), a downstream kinase of insulin signaling. In addition, Akt decreases INSIG2 mRNA and translocates the SCAP-SREBP-1c precursor complex from the endoplasmic reticulum to the Golgi. The mature SREBP-1c translocates into the nucleus, where this transcriptional factor binds to the sterol regulatory element (SRE) response element to activate the transcription of the lipogenic genes, including GPAT1. The metabolic products in GPAT1-mediated triacylglycerol (TAG) synthesis, including phosphatidic acid (PA) and diacylglycerol (DAG), reduce the insulin pathway, suggesting that GPAT1 is involved in insulin resistance (see below).

The most striking feature of GPAT1 transcription is the remarkable GPAT1 mRNA increase in fasting and refeeding [29]. The GPAT1 mRNA level increases more than 20-fold in the mouse liver when a fasted mouse is refed with a high-carbohydrate, fat-free diet. The fasting/refeeding-induced GPAT1 mRNA increase is mediated by the activation of SREBP-1c because fasted and refed mice reduce and raise the SREBP-1c mRNA level in the liver, respectively [30]. Fasting/refeeding-induced GPAT1 mRNA is not increased in the liver in streptozotocin-induced diabetic mice, whereas the injection of insulin into these mice increases the GPAT1 mRNA level in the liver [29]. The insulin-induced increase of GPAT1 mRNA results from the activation of SREBP-1c because insulin increases SREBP-1c mRNA in the livers of rats with streptozotocin-induced diabetes [31]. Insulin also activates LXR by increasing the levels of LXR ligands, oxysterols such as 24,25-epoxycholesterol, and 25-hydroxycholesterol. This activation of insulin leads to the increased transcription of SREBP-1c [32]. Reversely, dibutyryl cAMP has prevented the feeding-induced increase of GPAT1 mRNA in the liver, indicating that the glucagon-mediated elevation of cAMP negatively regulates the transcription of GPAT1 [29]. Thus, TAG synthesis in the liver is hormonally and nutritionally regulated by the SREBP-1c-related control of GPAT1 transcription, as shown in Figure 3. It has been known that LXR upregulates the transcription of enzymes related to hepatic lipogenesis other than GPAT1. LXR activates not only SREBP-1c but also the carbohydrate response element-binding protein (ChREBP). The synthetic LXR agonist T0901317 increases the hepatic mRNA of lipogenic enzymes, such as fatty acid synthase, by activation of ChREBP, whereas the increase of GPAT1 mRNA is promoted only by the activation of SREBP-1c [33]. Consequently, it is not likely that ChREBP contributes to the transcriptional regulation of GPAT1 in the liver.

**Figure 4:**
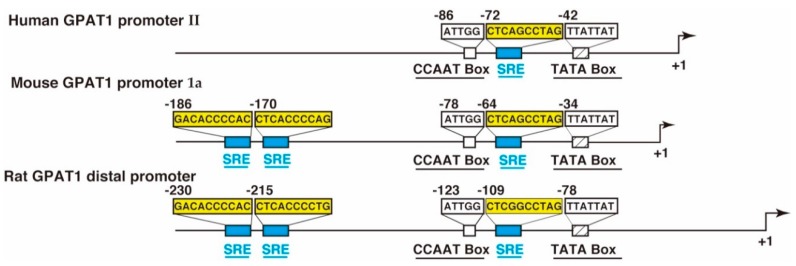
A schematic representation of the human acyl-CoA:glycerol-*sn*-3-phosphate acyltransferase1 (GPAT1) promoter II [39], the mouse GPAT1 promoter 1a [36], and the rat GPAT1 distal promoter [37]. The location of the sterol regulatory element (SRE) motif, the inverted CCAAT box, and the TATA box are shown. The number indicates the position from the transcription start site, which is designated as +1.

The promoter sequences of GPAT1 have been identified in mice [34,35,36], rats [37,38], and humans [38]. These species have two transcriptional sites and two promoters (mouse: Promoter 1a and promoter 1b; rat: Distal promoter and proximal promoter; human: Promoter I and promoter II). The promoter region corresponding to the mouse promoter 1a, the rat distal promoter, and the human promoter II is highly conserved, as shown in Figure 4. The sterol regulatory elements (SREs) recognized by sterol regulatory element-binding protein-1c (SREBP-1c) are located in these promoter regions with an inverted CCAAT box. 

The transcriptional activity of hepatocyte nuclear factors (HNFs) is examined in the human GPAT1 promoter II using HepG2 cells transfected with a transcriptional factor-encoding plasmid [39]. The overexpression of HNF1α slightly increases the GPAT1 promoter II activity. By contrast, HNF3α (Foxa1) decreases the human GPAT1 promoter II activity, which is consistent with the decrease in human GPAT1 mRNA levels in HNF3α-overexpressing human hepatocytes [40]. The overexpression of HNF3β (Foxa2) does not increase human GPAT1 promoter II activity, although HNF3β increases the hepatic GPAT1 mRNA levels in mice [41]. Furthermore, the HNF4α and CCAAT/enhancer binding of protein β (c/EBPβ) slightly activate the human GPAT1 promoter I. However, the effect of these transcription factors on the activation of the human GPAT1 promoter I or II is very low when compared with SREBP-1c. 

The promoter of GPAT1 is epigenetically regulated by DNA methylation [42]. The mouse neonatal liver slightly expresses GPAT1 mRNA, whereas the adult liver expression of GPAT1 mRNA is increased about 40-fold. This remarkable increase in GPAT1 transcription is caused by the demethylation of methylated cytidine in the CpG islands, flanking the three SRE elements upstream of the mouse GPAT1 promoter Ia. The decreased DNA methylation allows the recruitment of SREBP-1c to the SRE elements of the GPAT1 promoter; this is because the methylation of the GPAT1 promoter, which is induced by the CpG DNA methyltransferase (DNMT) 3b out of the three kinds of DNMTs, inhibits the recruitment of SREBP-1c [41].

As described above, the transcriptional regulation of GPAT1 has been well investigated, but few studies have addressed the other GPAT isoforms. It is likely that the transcription of the three GPAT isoforms is controlled by transcription factors other than SREBP-1c. The transcription of GPAT2 is up-regulated by retinoic acid (RA); because both 9-cis-retinoic acid and all-trans retinoic acid activate the transcriptional activity in mouse Sertoli cells, TM4 cells transfected with the GPAT2 promoter construct (−1324 to +2105) [43]. The predicted binding site for RA at +89 in the first intron might be involved in the RA response in the GPAT2 promoter [43]. The GPAT2 promoter contains 10 CpG islands at −92 to +32. The unmethylated GPAT2 promoter construct shows higher transcriptional activity than the methylated GPAT2, suggesting that GPAT2 is epigenetically regulated by DNA methylation, as well as by the GPAT1 promoter [42].

One of the transcriptional factor candidates to regulate the mRNA synthesis of another isoform (GPAT3) is a peroxisome proliferator-activated receptor γ (PPARγ), which is known to induce the expression of lipogenic genes [44]. The treatment of *ob*/*ob* mice with a PPARγ agonist, rosiglitazone, induces GPAT3 mRNA expression in WAT 4.5-fold [17]. GPAT3 mRNA is increased in calcium-induced human keratinocyte differentiation, whereas the mRNA levels of other GPAT isoforms remain unchanged [45]. This increase in GPAT3 results from the activation of PPARγ because the PPARγ agonist ciglitazone elevates mRNA synthesis in human keratinocytes about 3-fold [45]. PPARγ forms a heterodimer with RXR and binds to the DNA sequence’s designated peroxisome proliferator-activated receptor (PPAR) response elements (PPRE), flanking upstream of the target genes [46]. Although PPRE-like sequences are actually found upstream of the GPAT3 gene in both mice and humans, the involvement of these sequences in promotor activation remains undetermined.

### 3.2. Post-Translational Regulation

Insulin regulates GPAT1 not only at a transcriptional level but also at a post-transcriptional level in adipocytes. The treatment of rat adipose tissue mitochondria with insulin increases the Vmax (the maximum reaction rate) and Km (the substrate concentration at 1/2 Vmax) for both palmitoyl-CoA and G3P. The insulin-treated mitochondria show higher enzyme activity than the control mitochondria at a physiological G3P concentration lower than 1 mM. It is likely that these effects of insulin on the kinetic properties of GPAT1 are caused by the phosphorylation of two serine residues in GPAT1 [47]. In contrast, GPAT1 is negatively regulated by AMP-activated kinase (AMPK), which plays a key role in the control of energy metabolism [48]. AMPK activation decreases the TAG content through the inhibition of lipid synthesis and the stimulation of lipid oxidation. When AMPK is activated by an AMP-analog, 5-amino-4-imidazolecarboxamide (AICA) riboside (AICAR) is cultured in the rat hepatocyte and the TAG synthesis is reduced [49]. The inhibitory effect of AMPK on TAG synthesis is due to the decrease in GPAT1 activity because purified recombinant AMPK directly inhibits GPAT1 activity in a time and AMP-dependent manner [49]. AMPK phosphorylates and inactivates acetyl-CoA carboxylase (ACC), which catalyzes the formation of malonyl-CoA [50]. Malonyl-CoA results in decreased fatty acid oxidation because malonyl-CoA prevents the transport of long-chain fatty acids into the mitochondria by the inhibition of carnitine palmitoyltransferase I [51]. Thus, under excess energy conditions, AMPK-induced ACC inhibition and GPAT1 activation increase TAG synthesis and decrease the fatty acid oxidation [49]. In contrast to the acute effect of AMPK, when AICA is injected into the rat for six weeks, AMPK decreases the hepatic TAG accumulation without any difference in the hepatic GPAT1 activity [52]. Chronic AMPK activation downregulates hepatic TAG synthesis by a mechanism independent of a reduction in the GPAT1 activity [52]. 

## 4. Association of the GPAT Expression and Activity with Pathological and Physiological Functions

### 4.1. Insulin Resistance

Insulin resistance is a pathological condition, in which cells fail to take up glucose from the blood in response to insulin. Therefore, insulin resistance elevates blood glucose levels and increases the risk of the development of type 2 diabetes mellitus (T2DM). Several lines of evidence have demonstrated that insulin resistance is associated with the intracellular ectopic accumulation of TAG in non-adipose tissues, particularly in the liver [53]. Many studies indicate the involvement of mitochondrial GPAT1, which occupies 30%–50% of the total GPAT activity in the liver, in hepatic steatosis. It has been reported that hepatic steatosis is associated with insulin resistance and T2DM. Nagel et al. reported that the overexpression of GPAT1 in the rat liver caused hepatic steatosis and insulin resistance in the absence of obesity or high-fat feeding [54]. The adenovirus-mediated hepatic overexpression of GPAT1 remarkably increases the intracellular content of the metabolites of GPAT1, such as LPA and DAG [54]. The hypothesis that GPAT1 is involved in insulin resistance is supported by the study using GPAT1 ^−/−^ mice [55]. Consistent with the results obtained from the overexpression of GPAT1, GPAT1^−/−^ mice showed remarkably lower amounts of hepatic TAG and DAG and were prevented from high-fat feeding-induced hepatic steatosis and hepatic insulin resistance [55]. It is likely that insulin resistance is caused by the activation of protein kinase C*ϵ* (PKC*ϵ*), which is induced by the metabolite DAG that is produced in the process of TAG synthesis [56]. In fact, increased PKC*ϵ* activation and decreased PKC*ϵ* activity are observed in GPAT1 overexpressing mice and GPAT1 knockout mice, respectively [55]. Leptin-deficient *ob/ob* mice suffer from insulin resistance. In these mice, the GPAT1 activity and mRNA are increased [57]. Although the improvement of insulin resistance is also expected in *ob/ob* mice lacking GPAT1, the GPAT1 deficiency in *ob/ob* mice diminishes hepatic steatosis but does not protect against insulin resistance [57]. These results suggest that decreased hepatic steatosis alone does not improve insulin resistance and that a mechanism other than the DAG-induced PKC*ϵ* activation contributes to insulin resistance in *ob/ob* mice. As a putative mechanism, other than in PKC*ϵ* signaling, TAG synthesis is involved in insulin resistance mediated by mTOR complex 2 (mTORC2) [58]. mTOR is a Ser/Thr protein kinase that belongs to the phosphoinositide 3 kinase (PI3K)-related kinase family. mTOR exists in two multiprotein complexes, mTORC1 and mTORC2. mTORC2 forms a complex consisting of mTOR and a protein known as rictor. The overexpression of hepatocyte GPAT1 inhibits the insulin-stimulated phosphorylation of Akt, which is a substrate of mTORC2 and suppresses insulin signaling. The inhibition of mTOR/rictor binding and mTORC2 activity coincides with the levels of PA and DAG which contain palmitic acid. Above all, the PA and DAG produced by the glycerol phosphate pathway downregulate insulin action and the TAG synthesis is associated with hepatic insulin resistance. 

The proportion of hepatic GPAT activity is highest in mGPAT1, while microsomal GPAT3 and GPAT4 account for substantial GPAT activity [24]. GPAT4^−/−^ mice are protected from insulin resistance caused by high-fat feeding [55], as observed in GPAT1^−/−^ mice [59]. The overexpression of GPAT4 in mouse hepatocytes impairs insulin-suppressed gluconeogenesis and insulin-stimulated glycogen synthesis [55]. The mechanism by which the overexpression of GPAT4 impairs insulin-signaling is similar to that of GPAT1 [58]. The overexpression of GPAT4 blocks the insulin-stimulated phosphorylation of Akt by inhibition of the mTOR/rictor association and mTORC2 activity. Interestingly, the knockout and the overexpression of microsomal GPAT3 do not alter insulin signaling [59]. The insulin-stimulated phosphorylation of Akt is inhibited by PA, which is produced by GPAT3, and subsequent enzyme reactions. Mouse hepatocytes overexpressing GPAT4 contain a larger amount of dipalmitoyl PA, as compared with GPAT3. GPAT3 and GPAT4 might show the different inhibitory effect on insulin signaling due to the different molecular species of PA produced by the respective GPAT isoforms. 

### 4.2. Obesity

Obesity is a medical condition in which excess fat accumulates in the body. Obesity is associated with many human diseases, including T2DM, cardiovascular diseases, and cancer [60]. Physiologically, obesity is regarded as the result of the expansion of the WAT. Expansion of the WAT is primarily caused by the excess storage of TAG, when TAG synthesis exceeds TAG degradation [61]. 

Knockout and overexpression experiments have been used to examine whether the mitochondrial isoform GPAT1 is associated with obesity in murine models. GPAT1^−/−^ mice experience reductions in body weight and an increased liver TAG content when compared with wild-type mice, suggesting that GPAT1 is involved in both the obesity and dyslipidemia associated with obesity [62]. The knockout of GPAT1 expression in the liver of *ob*/*ob* mice reduces the hepatic TAG and DAG [63]. The hepatic TAG and DAG contain less palmitic acid [63], which is consistent with the findings obtained from GPAT1 knockout mice [62]. However, the hepatic overexpression of GPAT1 in rats shows no significant difference in body weight and fat pad weight, although augmented hepatic TAG synthesis causes hepatic steatosis [63]. Diet-induced obese and genetically obese mice experience an increase not only in the mitochondrial GPAT activity but also in the microsomal GPAT activity in the liver [64]. Above all, it is difficult to explain the obesity only by the increased hepatic GPAT1 activity. The proportion of the GPAT1 activity compared to the total GPAT activity is estimated to be about 10% in most tissues except the liver [1]. However, Morgan-Bathke et al. examined the GPAT1 activity in obese humans and reported that the GPAT1 activity accounted for 30%–60% of the total GPAT activity in both omental and abdominal subcutaneous adipose tissues [65]. Thus, GPAT1 activity in adipose tissue might contribute to the development of obesity, particularly in humans.

Microsomal isoform GPAT3 deficiency using knockout mice shows that GPAT3 accounts for predominant GPAT activity in WAT, which is closely related to obesity [66]. The total GPAT activity in subcutaneous WAT and visceral WAT is reduced by 80% and 50%, respectively. By contrast, the total GPAT activity in the liver remains unchanged [66]. GPAT3^−/−^ female mice, but not male mice, show a decrease in body weight and adiposity with increased energy expenditure [66]. The reason why there is the sex difference in the phenotypes of GPAT3^−/−^ mice remains unknown, although interactions of GPAT3 with sex hormones and their receptors are expected.

Another microsomal isoform, GPAT4, is also associated with obesity. GPAT4 deficiency in mice causes subdermal lipodystrophy, suggesting that GPAT4 plays a role in TAG accumulation in subdermal adipocytes [65]. GPAT4 deficiency in *ob*/*ob* mice decreases the TAG levels in BAT. GPAT4^−/−^-deficient mice exhibit a 25% reduction in body weight and a resistance to diet-induced and genetically-induced obesity with increased energy expenditure. The sex difference observed in GPAT3^−/−^ mice [64] has not been reported in GPAT4^−/−^ mice. 

### 4.3. Tumorigenesis

Non-alcoholic fatty liver disease (NAFLD) has been regarded as a risk of liver cancer [67]. Several mouse models show that hepatic steatosis increases the risk of hepatocellular carcinoma (HCC). For example, diet-induced and genetic-induced hepatic steatosis promote hepatic tumorigenesis in C57/Bl6 mice which are administered with pro-carcinogen diethylnitrosamine (DEN) as an inducer of HCC [68]. C57/Bl6 mice deficient in GPAT1, which mainly contributes to hepatic TAG accumulation, have reduced susceptibility to DEN-induced liver tumorigenesis [69]. Reactive oxygen species (ROS) are increased in the livers of GPAT1^−/−^ mice [69]. Furthermore, since phosphatidylethanolamine and phosphatidylcholine from GPAT1^−/−^ mice livers contain larger amounts of arachidonate instead of palmitate (the preferable substrate of GPAT1), GPAT1^−/−^ mice contain larger amounts of 4-hydroxynoenal (4-HNE), which is a peroxidation product of arachidonic acid [69]. Although the mechanism by which GPAT1 deficiency prevents liver tumorigenesis remains unknown, it is likely that the change in metabolic intermediates and the alteration in the molecular species of phosphatidylcholine and phosphatidylethanolamine by GPAT1 regulate the proliferation of hepatocytes through the apoptosis. 

On the other hand, glycerophosphocholine (GPC) phosphodiesterase (GPC-PD), which hydrolyzes GPC to choline and G3P, has been identified as a potential marker of metastasis in endometrial cancer [70]. In more recent reports, GPAT1, which utilizes the GPC-PD product G3P as an enzyme substrate, promotes tumor cell migration and tumor cell growth in ovarian carcinoma [71]. The knockout of GPAT1 reduces the cell migration in MCF7 and HeLa cells, whereas choline kinase-α, which utilizes another GPC-PD product (choline) as an enzyme substrate, reveals no effect on cell migration [71]. GPAT1-induced cell migration is associated with an increase of LPA, which reveals the cell proliferation and migration activities [72] because the knockout of GPAT1 decreases intracellular palmitoyl and oleoyl-LPA levels [71]. An increased LPA level has been reported to be a potential marker for ovarian cancer in particular. The meta-analysis of ovarian cancer databases shows that higher GPAT1 expression is significantly associated with shorter overall survival [70]. LPA levels in plasma and ascites are significantly higher in ovarian cancer patients than in control subjects [73]. In addition to GPAT1, LPA is produced by the hydrolysis of polyunsaturated lysophosphatidylcholines [73]. This reaction is catalyzed by a secreted enzyme, lysophospholipase D [6]. Autotaxin, which was originally identified as a tumor cell motility-stimulating factor, has lysophospholipase D activity [6].

The cancer-testis (CT) gene encodes for a protein which is expressed not only in the testes but also in tumors [74]. GPAT2 is proposed to be a new member of the CT genes which contribute to tumorigenesis and metastasis [75]. Recently, Pellon-Maison et al. have reported that GPAT2 is highly expressed in human cancers like melanoma, lung, prostate, and breast cancer, and promotes a malignant phenotype [76]. The GPAT2 expression pattern in different tumors is consistent with other CT genes in human cancer. The knockout of GPAT2 in the breast cancer cell line (MDA-MB-231 cells) markedly diminishes proliferation and migration [76]. Furthermore, the knockout of GPAT2 dramatically increases staurosporine-induced apoptosis [76]. Consistent with these in vitro results, the inoculation of GPAT2-deficient breast cancer cells fail to grow in the nude mice. Interestingly, GPAT2-silenced MDA-MB-231 cells alter the integrity of the cellular membrane, suggesting that this increased membrane permeability inhibits the proliferation of GPAT2-silenced MDA-MB-231 cells [77]. piRNA is a member of the small non-coding RNAs (sncRNAs) and interacts with P-element-induced wimpy testis (Piwi) proteins. GPAT2 is involved in the biosynthesis of piRNA [78]. The expression of piRNA is associated with the development of cancer [79,80], although the mechanism by which piRNA causes tumorigenesis is unknown. Very recently, the same group further found that nine micro-RNAs (miRNAs), which are usually upregulated in breast cancer and are associated with a worse survival prognosis, are downregulated in the GPAT2-silenced MDA-MB-231 cells [81]. Above all, GPAT2 might be a new target of cancer therapy. 

### 4.4. Spermatogenesis

GPAT2, which is highly expressed in testes [14], is necessary for normal spermatogenesis. GPAT2 is bound to the MILI protein, one of the three mouse Piwi proteins, and the knockout of GPAT2 impairs the production of the Piwi-interacting protein, piRNA, in germline stem cells [78]. It is known that piRNA prevents the expression of deleterious retrotransposons, thereby ensuring genome integrity during meiosis [82]. GPAT2 mRNA and protein expression reach a peak at 15 days post-partum (dpp), when the pachytene spermatocytes predominate, which correlates with piRNA synthesis [43]. The acyltransferase activity of GPAT2 is not necessary for piRNA synthesis because mutant GPAT2, which lacks Motif I, promotes the piRNA production [78]. GPAT2 silencing in male mice shows impaired spermatogenesis and arrest at the pachytene stage [83]. Furthermore, the reproductive capacity of these mice is reduced and they express an apoptosis-related gene [83]. The TAG content in the GPAT2-silencing mice does not vary between GPAT2-silencing mice and control mice, suggesting that acyltransferase activity is not necessary for normal spermatogenesis. 

## 5. Conclusion and Future Directions

The dramatic elevation of TAG synthesis in fasting/refeeding is nowadays explained by transcriptional regulation of GPAT1, which is first identified in DNA and at a protein level. Its promoter sequence, which interacts with the potent transcription factor SREBP-1c, is identified and it is shown that transcription is epigenetically regulated through the methylation and demethylation of the CpG islands flanking in the promoter. It is not likely that SREBP-1c, which is commonly known as a transcription factor in lipogenic tissues, is associated with the other three GPAT isoforms. RA and PPARγ are predicted as putative transcription factors for GPAT2 and GPAT3, respectively, but the detailed structure of their promoters, including the DNA sequence which interacts with these transcription factors, remains undetermined. Progress in this field will open the gate to understanding the association of GPAT isoforms with physiological and pathophysiological functions, including obesity and tumorigenesis, in the future. 

In this review, we focused on the transcriptional regulation of GPATs. Although GPATs are the key enzymes and are involved in the rate-limiting step of TAG synthesis, other enzymes involved in the sequential steps are also known to be transcriptionally regulated. For example, the mRNA of AGPAT2, which catalyzes the second step of TAG synthesis, is abundantly expressed in adipose tissues [84]. The expression of AGPAT2 mRNA is increased during adipocyte differentiation, suggesting that AGPAT2 transcriptionally controls TAG synthesis in the adipose tissues. It has been reported that inactivating the mutation of AGPAT2 causes congenital generalized lipodystrophy. As seen in this case, it will be necessary to elucidate the linkage of GPATs and other related enzymes in the various tissues which store TAG, particularly by transcriptional levels. 

It should be noted that glycerol phosphate pathway enzymes, including GPATs, are also therapeutic targets because excess TAG storage is associated with insulin resistance, obesity, and tumorigenesis. Several inhibitors to GPATs have been reported to date for clinical use [85]. However, these compounds inhibit all four GPAT isoforms because the inhibitors are designed based on the conserved Motif I, which is common in all four GPAT isoforms. It is expected that inhibitors selective for each GPAT isoform will be designed and used for therapy.
